# Copper excess reduces nitrate uptake by *Arabidopsis* roots with specific effects on gene expression

**DOI:** 10.1016/j.jplph.2018.06.005

**Published:** 2018-09

**Authors:** Franz W.R. Hippler, Dirceu Mattos-Jr, Rodrigo M. Boaretto, Lorraine E. Williams

**Affiliations:** a Centro de Citricultura Sylvio Moreira, Instituto Agronômico (IAC), Rod. Anhanguera, km 158, CP 04, CEP 13490-970, Cordeirópolis, SP, Brazil; b University of Southampton, Biological Sciences, Building 85, Highfield, Southampton SO17 1BJ, United Kingdom

**Keywords:** HAT, high-affinity transporters, LAT, low-affinity transporters, LOX, lipoxygenase, ROS, reactive oxygen species, Nitrate transporter, Proton pump, Metal toxicity, Nutrient uptake

## Abstract

Nitrate uptake by plants is mediated by specific transport proteins in roots (NRTs), which are also dependent on the activity of proton pumps that energize the reaction. Nitrogen (N) metabolism in plants is sensitive to copper (Cu) toxicity conditions. To understand how Cu affects the uptake and assimilation processes, this study assesses the inhibitory effects of elevated Cu levels on the expression of genes related to N absorption, transport and assimilation in roots of *Arabidopsis*. Plants were grown hydroponically for 45 days, being exposed to a range of Cu concentrations in the last 72 h or alternatively exposed to 5.0 μM Cu for the last 15 days. High Cu levels decreased the uptake and accumulation of N in plants. It down-regulated the expression of genes encoding nitrate reductase (*NR1*), low-affinity nitrate transporters (NRT1 family) and bZIP transcription factors (*TGA1* and *TGA4*) that regulate the expression of nitrate transporters. Cu toxicity also specifically down-regulated the plasma membrane proton pump, *AHA2*, whilst having little effect on *AHA1* and *AHA5*. In contrast, there was an up-regulation of high-affinity nitrate transporters from the NRT2 family when exposed to medium level of Cu excess, but this was insufficient for restoring N absorption by roots to control levels. These results demonstrate that plants display specific responses to Cu toxicity, modulating the expression of particular genes related to nitrate uptake, such as low-affinity nitrate transporters and proton pumps.

## Graphical abstract

**Figure:**
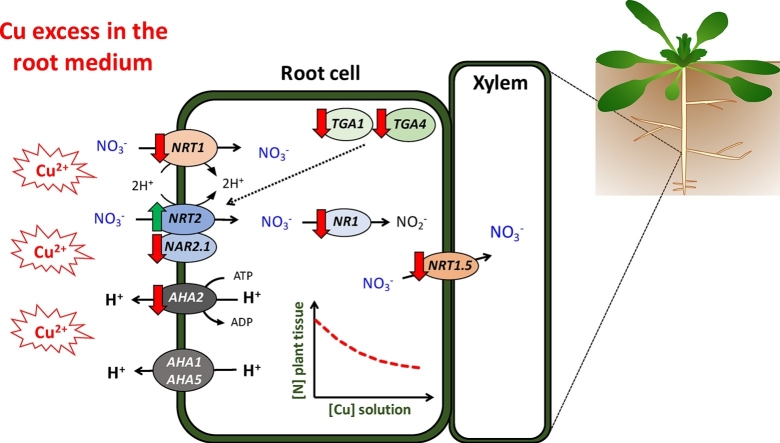

## 1 Introduction

Nitrogen (N) is a key element for plants and its availability is a major factor determining plant growth and crop production. Specific transporters are important in N acquisition by plant roots and contribute to N use efficiency (Williams and Miller, 2001; Araki and Hasegawa, 2006). Among the inorganic N-forms, nitrate is the main source in aerobic soils (Crawford and Glass, 1998). Nitrate uptake across the plasma membrane of root cells occurs through specific transporters categorized as the NRT1/PTR family (NPF), “low-affinity” transporters (LAT), and the NRT2, “high-affinity” transporters (HAT) (Wang et al., 2012). The LAT genes are highly expressed in high (>0.5 mM) external nitrate concentration, while HAT genes are expressed when external nitrate concentration in the medium is low (≤0.5 mM) (Wang et al., 2012; Fan et al., 2017). In *Arabidopsis*, fifty-three genes were characterised that encode LAT genes and at least seven genes encoding the HATs (Wang et al., 2012).

The activity and transcription of genes that encodes nitrate transporters are dependent also on other genes. *NAR2.1* (nitrate assimilation related protein; also known as *NRT3.1*) interacts with NRT2 and works together as a HAT of nitrate (Kotur et al., 2012). In *Arabidopsis*, six of the seven NRT2 family members require *NAR2.1* for transporting nitrate across the plasma membrane. The exception is *NRT2.7*, which controls nitrate concentration in seeds, and is thought not to require *NAR2.1* (Kotur et al., 2012). *TGA1* and *TGA4* encode bZIP-transcription factors that are candidate regulatory factors mediating nitrate responses (Alvarez et al., 2014). *TGA1* and its close homolog *TGA4* were shown to participate in plant defence responses against pathogen and chemical stress (Gatz, 2013), and are induced in response to nitrate treatments in *Arabidopsis* roots (Alvarez et al., 2014). They are also important in pathways leading to root hair development in response to nitrate (Canales et al., 2017).

Plasma membrane (PM) H^+^-ATPases also play a role in the absorption of N by energizing transport and creating a proton gradient (H^+^) between the cytosol and apoplast (Camacho-Cristóbal and González-Fontes, 2007; Sperandio et al., 2011). The absorption of nitrate depends on H^+^ pumping, which requires the entry of 2H^+^ for each nitrate actively transported across the PM (Chapman and Miller, 2011). In *Arabidopsis* there are eleven members of AHA family (*AHA1*-*AHA11*; Młodzińska et al., 2015). *AHA1* and *AHA2* are genes expressed at high levels in *Arabidopsis* by a range of stimuli (Haruta et al., 2010). *AHA2* is the predominant proton pump in roots and it is upregulated after nitrate supply (Młodzińska et al., 2015). In rice (*Oryza sativa*), the expression of the genes *OsA1*, *OsA2* and *OsA5* were reduced in roots by nitrate-starvation in the root medium (Sperandio et al., 2011).

Copper (Cu) is also an essential element for plant growth, required in trace amounts (Hall and Williams, 2003; Mikkelsen et al., 2012; Yruela, 2013). In excess it causes oxidative stress, with increased production of reactive oxygen species (ROS) via the Haber-Weiss reaction and consequent peroxidation of lipid components of cell membranes (Hall, 2002; Ravet and Pilon, 2013). Excess Cu increases the activity of lipoxygenase (LOX) enzyme that also catalyse the peroxidation of lipids, mainly using free fatty acids as substrates (Newcomer and Brash, 2015; Cuypers et al., 2016). High Cu levels also inhibit the synthesis of proteins and their function with irreversible linkages established between the metal and sulfhydryl groups. As an overall process, photosynthesis rate decreases (Drzewiecka et al., 2017; Hippler et al., 2018) and plant growth is affected (Mattos Jr et al., 2010). Nutritional disorders caused by Cu excess include an interference in the reduction of nitrate to ammonium, primarily by inhibiting the activity of nitrate reductase. In *Citrus* this subsequently impairs the incorporation of N into proteins, leading to the accumulation of nitrate in plant tissues (Hippler et al., 2016). In grape vine (*Vitis vinifera*) excess Cu also impairs the absorption of nitrate by roots, but accumulation of nitrate did not occur in this case despite a reduced nitrate reductase activity (Llorens et al., 2000). Elevated Cu also causes inhibition of the PM H^+^-ATPase in tomato (*Solanum lycopersicum*; Zhang et al., 2009) but the underlying mechanisms involved are not certain. The intensity of metal stress effects on H^+^-ATPase activity depends on the type and concentration of the heavy metal and the length of the exposure time of roots (Janicka-Russak et al., 2012).

Cu-contaminated soils occur world-wide due to the intensive applications of cupric based fungicides; therefore it is important to assess how Cu excess affects plant responses, in particular the acquisition and assimilation of nitrate, the main source of inorganic-N to plant roots. In this study, we investigated the effects of elevated Cu on the expression of genes related to nitrate uptake, transport and assimilation in roots of *Arabidopsis* and demonstrate specific responses to Cu toxicity.

## 2 Materials and methods

### 2.1 Plant growth

**Fig. 1:**
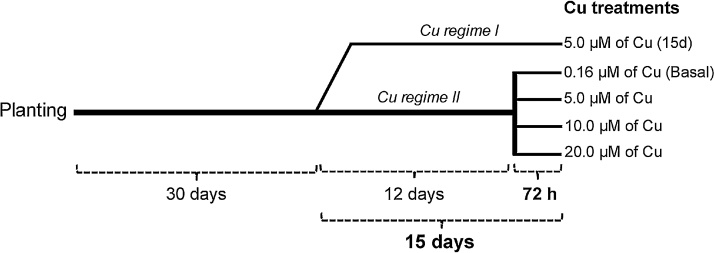
Schematic time line showing experimental treatments for *Arabidopsis thaliana* grown in nutrient solution with varying copper (Cu) concentrations up to 72 h or 15 days (15d). During the first 30 days of the experiment, plants received 0.16 μM Cu as a basal condition.

Sterilised seeds of *Arabidopsis thaliana* (L.) Heynh Columbia (Col-0) were placed individually in plastic tubes (0.5 mL) containing 0.5% (w/v) agar, stored in the dark at 4 °C for 48 h and then transferred into a controlled-environment growth room (23 °C, 8 h light: 120 μmol photons m^−2^ s^–1^/ 23 °C, 16 h dark cycle). Seedlings were grown in hydroponic conditions with half-strength concentration of a control nutrient solution for 15 days and, subsequently with full-strength concentration. The control nutrient solution was modified from Hoagland, and contained 1.25 mM KNO_3_, 0.5 mM Ca(NO_3_)_2_·4H_2_O, 0.5 mM MgSO_4_·7H_2_O, 0.625 mM KH_2_PO_4_, 2.0 mM NaCl; 42.5 μM FeNa-EDTA, 0.38 μM ZnSO_4_·7H_2_O, 1.8 μM MnSO_4_·7H_2_O, 45.0 μM H_3_BO_3_, 0.015 μM (NH_4_)_6_Mo_7_O_24_·4H_2_O, 0.01 μM CoCl_2_ and 0.16 μM CuSO_4_·5H_2_O, adjusted to pH 5.6 using 0.5 M KOH (Nazri et al., 2017). Thirty days after planting, a set of plants were grown as indicated in Fig. 1 under Cu regime I. This was with the same control nutrient solution but containing 5.0 μM Cu, for a further 15 days (Fig. 1). Another set of plants, continuously growing with the control nutrient solution (42 days with full-strength concentration), were grown under Cu regime II with varying Cu concentrations (5.0, 10.0 or 20.0 μM Cu), for 72 h (Fig. 1). In all growing conditions, nutrient solutions were changed weekly. Additionally, control plants were also grown with the nutrient solution containing 0.16 μM Cu (Basal; Fig. 1). Each Cu treatment had 15 plants and was replicated three times. Root samples were collected at 6, 24 and 72 h after initiation of the Cu treatments in liquid N and stored at −80 °C for gene expression analysis.

### 2.2 RNA extraction and Real-time PCR

Total RNA was extracted using TRIzol Reagent (Invitrogen Life Technologies, Carlsbad, CA, USA), and reverse transcription using the SuperScript system (Invitrogen) and oligo(dT) primer was carried out according to the manufacturer’s instructions. Real-time PCR was performed to measure gene expression was carried out as previously described (Jaffé et al., 2012; Nazri et al., 2017) using SYBR Green (Finnzymes) and an Opticon DNA Engine Continuous Fluorescence Detector (GRI Ltd.). PCR was performed using specific forward and reverse primers for each gene (Supporting information Table S1) at 95 °C for 10 min followed by 35 cycles of 95 °C for 15 s and 60 °C for 1 min. All gene expression analysis was performed with at least three independent biological replicates and the reactions were set up in triplicate for each sample. All data were standardized by normalizing to *SAND* or *Yellow-Leaf-Specific gene8* (*YLS8*) expression (Remans et al., 2008; Jaffé et al., 2012) and analysed using Opticon Monitor III software (Biorad). Quantification of the relative transcript levels was performed using the comparative Ct (threshold cycle) method.

### 2.3 Plants growth and nutritional status

Plants were collected and separated into shoots and roots and dried at 58–60 °C to constant dry weight. Samples were ground and nutrient content were measured by nitro-perchloric digestion according to Bataglia et al. (1983) by plasma emission spectrometry (ICP-OES, Perkin-Elmer 5100 PC, Norwalk, CT, USA).

### 2.4 Statistical analyses

Analysis of variance (ANOVA) was used to evaluate the results with a level of significance of α = 0.05. Effects of treatments were compared using Tukey test at 5%.

## 3 Results

### 3.1 Biomass production and nutritional status of plants

**Fig. 2:**
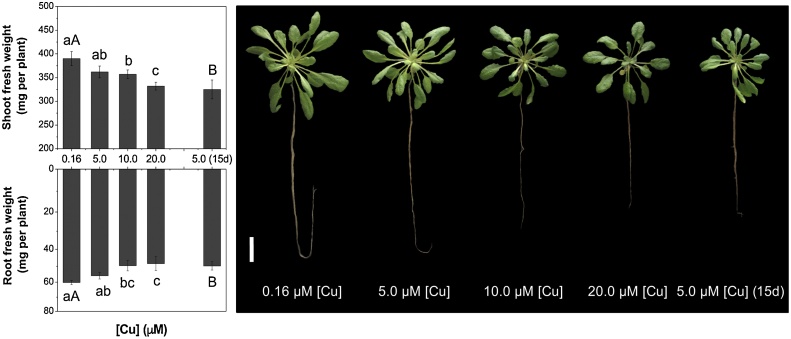
Elevated copper (Cu) negatively impacts fresh weight of shoots and roots of *Arabidopsis thaliana*. Plants were grown under hydroponic conditions for up to 72 h or 15 days (15d). Mean (+/– SEM) are shown (n = 3). For the Cu treatments for 72 h different lowercase letters indicate mean values are significantly different among the [Cu] (0.16, 5.0, 10.0 and 20.0 μM) by Tukey’s test (*p* < 0.05). For the Cu treatments for 15 days different uppercase letters indicate mean values are significantly different between the [Cu] (0.16 and 5.0 μM) by Tukey’s test (*p* < 0.05). White line in the image represent 2 cm.

**Fig. 3:**
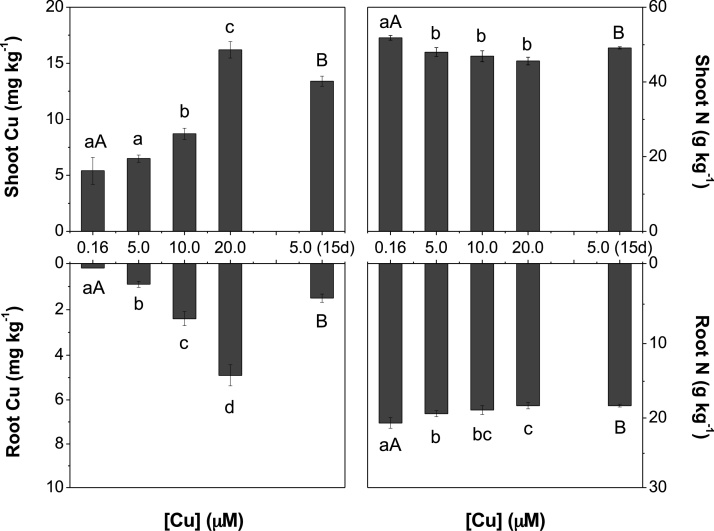
Elevated copper in the media (Cu) negatively affected nitrogen (N) and Cu concentrations in shoot and root of *Arabidopsis thaliana.* Plants were supplied with different Cu levels in the nutrient solution for 72 h or 15 days (15d). Mean (+/– SEM) are shown (n = 3). For the Cu treatments for 72 h different lowercase letters indicate mean values are significantly different among the [Cu] (0.16, 5.0, 10.0 and 20.0 μM) by Tukey’s test (*p* < 0.05). For the Cu treatments for 15 days different uppercase letters indicate mean values are significantly different between the [Cu] (0.16 and 5.0 μM) by Tukey’s test (*p* < 0.05).

The effect of two Cu regimes was determined in Arabidopsis. Forty-five day-old plants were assessed following exposure to varying Cu levels for the final 72 h or to 5.0 μM Cu for the final fifteen days (Fig. 1). No marked visual symptoms of Cu toxicity were observed in these plants at the concentrations tested (Fig. 2); however, root biomass decreased in the plants exposed to 10.0 and 20.0 μM Cu for 72 h and there was a reduction in shoot and root fresh weight for plants exposed to 5.0 μM Cu after 15 days (Fig. 2). Here we show that when exposed to Cu concentrations in the nutrient solution at ≥5.0 μM Cu, the concentration of this metal increased whereas N levels decreased both in shoot and root tissues (Fig. 3). Furthermore, the elevated levels of Cu in the nutrient solution also reduced the concentration of phosphorous (P), calcium (Ca), iron (Fe), manganese (Mn) and boron (B) in the shoots and Mn in the roots (Table 1). However, Fe concentration in roots increased with the higher Cu levels in the nutrient solution (Table 1). No changes were observed in concentrations of the potassium (K), magnesium (Mg), sulphur (S) and zinc (Zn) after Cu treatments (data not shown).

**Table 1:** Elevated copper (Cu) affected the concentration of phosphorus (P), calcium (Ca), iron (Fe), manganese (Mn) and boron (B) in the shoots and Fe and Mn in the root of *Arabidopsis thaliana* supplied with different Cu levels in the nutrient solution for 72 h or 15 days (15d).

Cu concentrations	Shoot						Root	
	P	Ca	Fe	Mn	Zn	B	Fe	Mn
μM	g kg^−1^	g kg^−1^	mg kg^−1^	mg kg^−1^	mg kg^−1^	mg kg^−1^	mg kg^−1^	mg kg^−1^
0.16	6.7 a^a^A^b^	30 aA	114 aA	115 aA	31 aA	31 aA	19 aA	1.6 aA
5.0	6.7 a	26 ab	95 b	113 a	30 a	28 a	31 b	1.3 ab
10.0	6.5 a	24 b	94 b	102 ab	29 a	29 a	29 b	0.7 bc
20.0	5.5 b	23 b	95 b	85 b	23 b	22 b	32 b	0.4 c

5.0 (15d)	6.3 A	25 B	84 B	90 B	30 A	31 A	29 B	0.2 B

a For the Cu treatments for 72 h different lowercase letters indicate mean values are significantly different among the [Cu] (0.16, 5.0, 10.0 and 20.0 μM) by Tukey’s test (*p* < 0.05).

b For the Cu treatments for 15 days different uppercase letters indicate mean values are significantly different between the [Cu] (0.16 and 5.0 μM) by Tukey’s test (*p* < 0.05).

### 3.2 Excess Cu alters the expression levels of nitrate transporter genes in roots

**Fig. 4:**
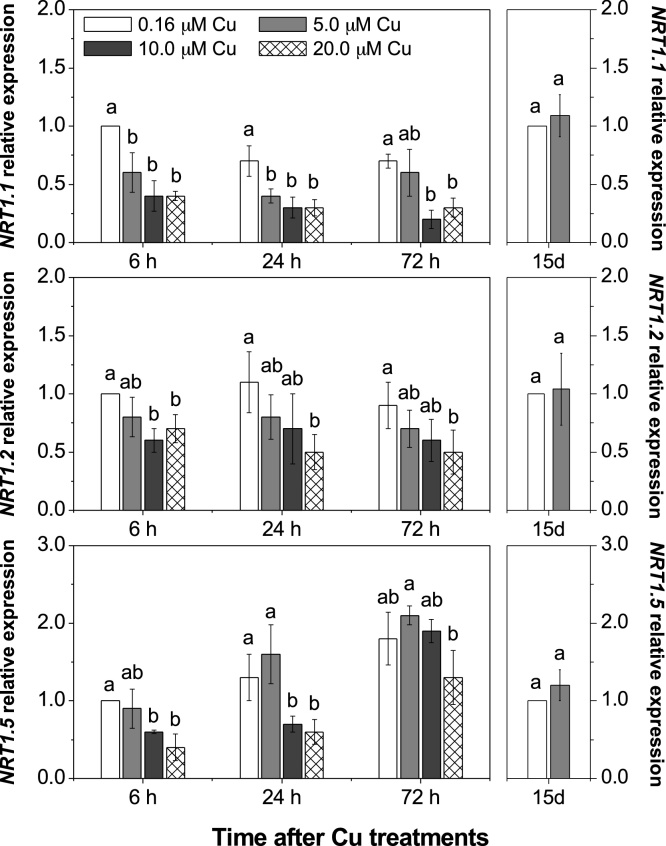
Elevated Cu negatively affected expression of low-affinity nitrate transporter (NRT1 family). Roots of *Arabidopsis thaliana* were supplied with different Cu levels in the nutrient solution for 72 h or 15 days (15d). Mean (+/– SEM) are shown (n = 3). Different letters in the same period are significantly different among the [Cu] (Tukey’s test, *p* < 0.05).

**Fig. 5:**
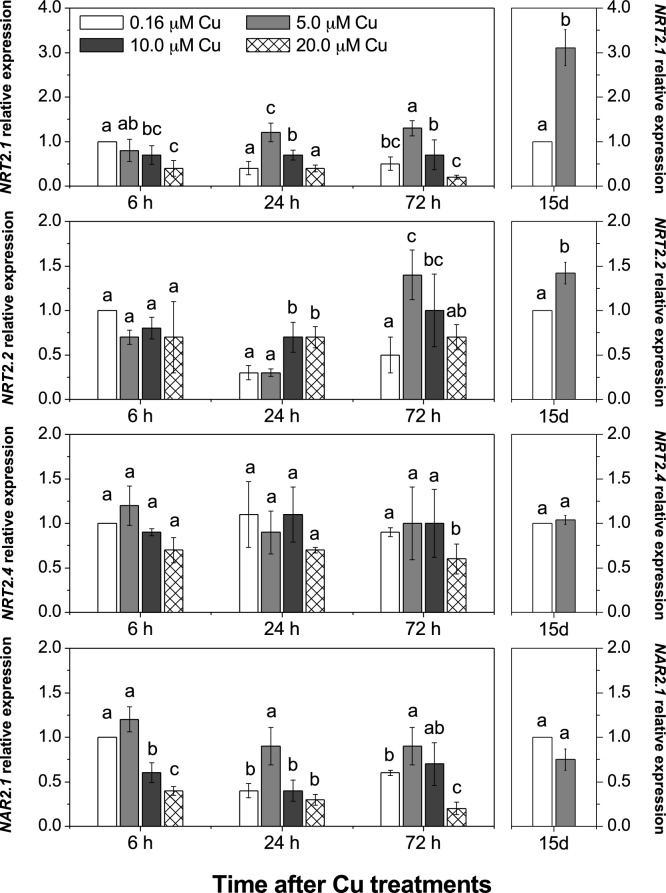
Elevated Cu up-regulated the high-affinity nitrate transporter (NRT2 family and NAR2.1). Roots of *Arabidopsis thaliana* were supplied with different Cu levels in the nutrient solution for 72 h or 15 days (15d). Mean (+/– SEM) are shown (n = 3). Different letters in the same period are significantly different among the [Cu] (Tukey’s test, *p* < 0.05).

The expression levels of genes encoding low and high-affinity transporters of nitrate in roots were analysed in response to Cu treatment. A decrease in *NRT1.1* (also named *NPF6.3* or *CHL1*; Fan et al., 2017) gene expression was observed when Cu increased ≥5.0 μM in the nutrient solution, whereas the expression of *NRT1.2* gene decreased for 10.0 up to 6 h or 20.0 μM Cu up to 72 h (Fig. 4). Furthermore, increased Cu concentration resulted in down-regulation of *NRT1.5* (Fig. 4), responsible for the transport of nitrate from the roots to the xylem vessels and consequently to the shoots. In contrast, for the HAT genes, *NRT2.1* and *NRT2.2,* there was an up-regulation in plants with 5.0 or 10.0 μM Cu after 24 h for *NRT2.1* and after 72 h for *NRT2.2*, both compared to basal Cu concentration in the nutrient solution (Fig. 5). *NRT2.1* and *NRT2.2* also showed an up-regulation following the 15 days Cu treatment at 5.0 μM Cu*. NRT2.4* is also classified as a HAT gene, which is reportedly up-regulated before *NRT2.1* and *NRT2.2* when plants are grown under nitrate deficiency (Wang et al., 2012). In this study, plants grown with 20.0 μM Cu exhibited a down-regulated expression after 72 h, while no change was observed for other Cu levels tested (Fig. 5). *NAR2.1* (also known as *NRT3.1*) encodes a protein required for the activation of *NRT2* members (Fig. 5). This showed a similar level of expression to *NRT2.1* apart from the 15 days Cu regime where in contrast to *NRT2.1* it was not markedly upregulated.

### 3.3 Specific effects of copper excess on transcript levels of H^+^-ATPase genes

**Fig. 6:**
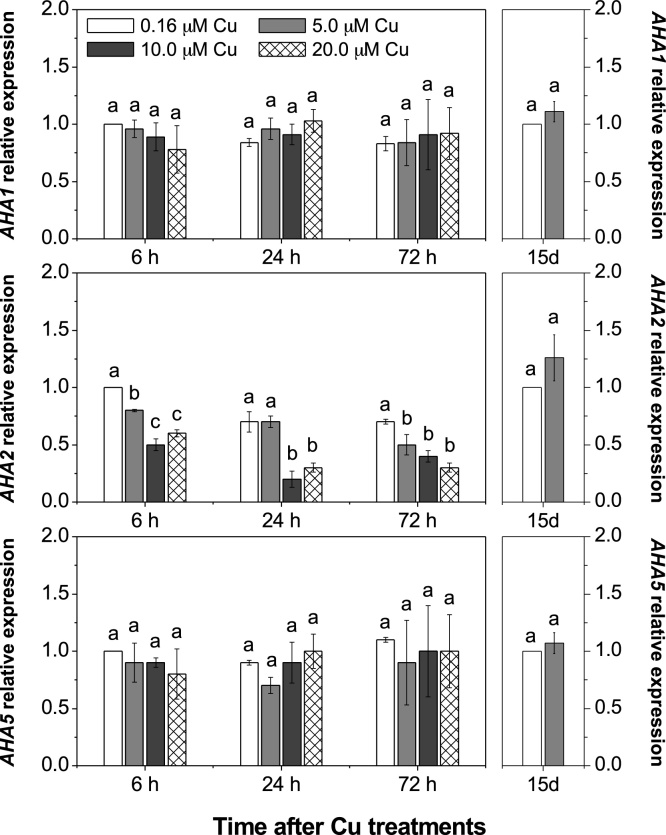
Effect of elevated Cu on H^+^-ATPase genes (AHA family). Roots of *Arabidopsis thaliana* were supplied with different Cu levels in the nutrient solution for 72 h or 15 days (15d). Mean (+/– SEM) are shown (n = 3). Different letters in the same period are significantly different among the [Cu] (Tukey’s test, *p* < 0.05).

Nitrate taken up by roots does not depend only on the NRT transporters, but also on H^+^-ATPases at the plasma membrane to energize the process and maintain the equilibrium of protons and anions in the cell. Here we have evaluated the expression of *AHA1*, *AHA2* and *AHA5*, after plants were exposed to different Cu concentrations, since these are considered among the most important genes transcribed in relation to nitrate uptake (Sperandio et al., 2011). In this study, only *AHA2* expression was specifically affected and this was seen to be down-regulated by Cu excess throughout the shorter time course regime (Fig. 6).

### 3.4 Nitrate reductase, transcription factors and lipoxygenase

**Fig. 7:**
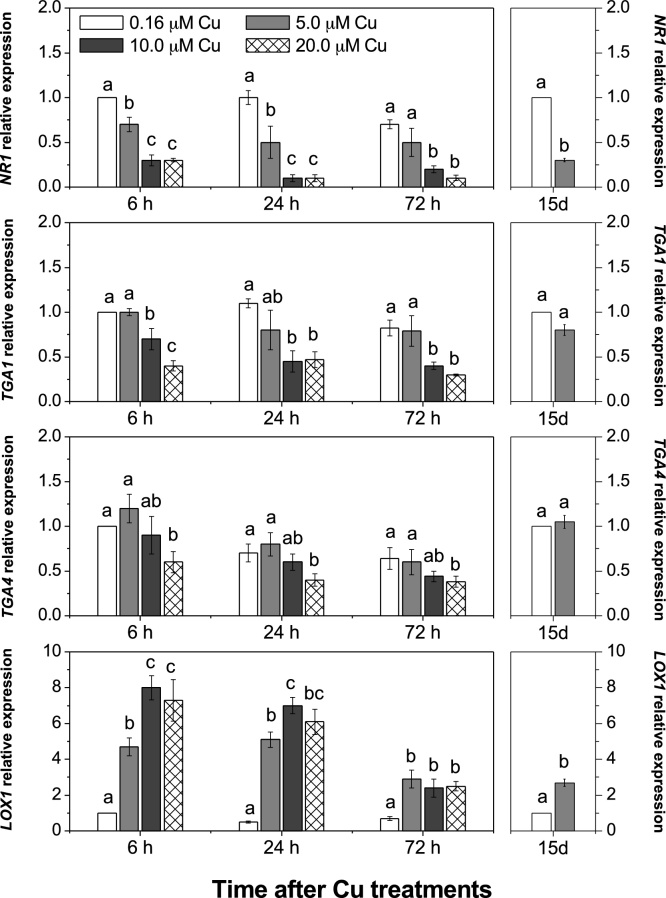
Effect of elevated Cu on the expression of genes encoding nitrate reductase (*NR1*), transcription factors (*TGA1* and *TGA4*) and lypoxygenase (*LOX1*). Roots of *Arabidopsis thaliana* supplied with different Cu levels in the nutrient solution for 72 h or 15 days (15d). Mean (+/- SEM) are shown (n = 3). Different letters in the same period are significantly different among the [Cu] (Tukey’s test, *p* < 0.05).

Nitrate reductase gene expression (*NR1*) was also highly affected by increasing Cu concentrations in the nutrient solution (Fig. 7). A decrease in *NR1* gene expression was observed under both Cu regimes. The bZIP transcription factors, *TGA1* and *TGA4,* which are reported to influence the expression of *NRT2.1* and *NRT2.2* in response to nitrate treatments (Alvarez et al., 2014) were down-regulated in roots of plants exposed to Cu excess (Fig. 7). *TGA1* gene expression was reduced when plants were exposed to 10.0 and 20.0 μM Cu, whereas *TGA4* occurred only for 20.0 μM, both after 6 h, 24 h and 72 h of metal exposure (Fig. 7).

*LOX1,* codes for an enzyme identified as lipoxygenase (LOX). Consistent with previous investigations (Cuypers et al., 2011), *LOX1* was markedly up-regulated by elevated Cu. This was observed under both Cu regimes used in this study (Fig. 7).

## 4 Discussion

Soil contamination with heavy metals and plant responses under abiotic stress conditions have been intensively studied. Accumulation of Cu in soil is a problem after intensive use of cupric fungicides that are used to preventively control diseases in many crops such as tomato (Rooney et al., 2006), vines (Ruyters et al., 2013) and citrus (Hippler et al., 2016). Excess Cu has previously been reported to reduce plant development and growth due to deleterious effects of this metal on protein and enzyme functions, as well as increasing reactive oxygen species (ROS) that impair physiological and biochemical processes such as photosynthesis (Drzewiecka et al., 2017). An increase in Cu availability in soils up to toxic levels is also followed by nutritional disorders affecting uptake and assimilation processes. N is often one of the most affected nutrients in plants and this can have implications on yield (Mattos Jr et al., 2010; Drzewiecka et al., 2017). Therefore, an understanding of the mechanism by which nutritional disorders occur in response to Cu toxicity is important.

In this study, we have investigated the effects of Cu toxicity in relation to N uptake and assimilation in more detail in roots of *Arabidopsis*. The threshold of Cu concentration that better demonstrated the specific effects of the metal excess on N absorption and assimilation processes were observed for those plants exposed to 5.0 μM and 10.0 μM Cu (Fig. 3; Supplementary File Fig. S1). Under 20.0 μM Cu, metal concentration increased in plants up to 3-fold in shoot and 25-fold in root (Fig. 3), both when compared to basal level, which resulted in plant growth inhibition (Fig. 2; Supplementary File, Fig. S2) and down-regulation of genes related to N absorption and assimilation, evaluated in this study (Fig. 4, Fig. 5, Fig. 6, Fig. 7).

In the first 72 h exposed to Cu excess, plants exhibited a reduction in the expression of *NRT1.1* and *NRT1.2,* representing the LAT of nitrate in roots (Fig. 3). These genes are important transporters responsible for nitrate uptake in roots when the nitrate concentration in the solution is adequate (≥0.5 mM; Wang et al., 2012), which was the case in this study. Increased Cu concentrations also resulted in down-regulation of *NRT1.5* (Fig. 4), which regulates long distances transport of nitrate into the xylem vessels and consequently to the shoots (Lin et al., 2008; Tegeder and Masclaux-Daubresse, 2017). Down-regulation of *NRT1.5* in *Arabidopsis* has been suggested as a tolerance mechanism for abiotic stress, in which nitrate is reallocated to plant roots (Chen et al., 2012; Goel and Singh, 2015). The *nrt1.5 Arabidopsis* mutant exhibited higher tolerance to either Cd, salt or drought stresses compared to wild type plants, which suggests that nitrate reallocation in roots contributes essentially to stress tolerance (Chen et al., 2012). Regardless of the down-regulation of the LAT genes (Fig. 4), plants exhibited an up-regulation of *NRT2.1* and *NRT2.2* after exposure to 5.0 and 10.0 μM Cu after 72 h and after 15 days of metal exposure for 5.0 μM Cu (Fig. 5). Under nitrate deficiency it has previously been reported that NRT1 genes are down-regulated while NRT2 genes are up-regulated to recover the nitrate from the solution (Araki and Hasegawa, 2006; Sperandio et al., 2011; Lupini et al., 2016). These results demonstrate that plants under medium Cu excess (not observed for 20.0 μM Cu) up-regulated HAT genes (Fig. 5) as an attempt to recover N uptake capacity, although this was not sufficient to restore the N content of plants (Fig. 3).

An accumulation of auxins and cytokinins in roots was observed in *Arabidopsis* exposed to Cu toxicity, which also influenced root architecture, resulting in low growth of primary roots and death of the root apical meristem (Lequeux et al., 2010). Changes in the expression of the NRT transporters could also affect hormonal signalling and transport (Chiba et al., 2015). Further understanding on the effect of Cu toxicity inhibiting the transport of some hormones due the down-regulation of NRTs genes is still required. *NRT1.1* expression modulates auxin levels in meristematic regions of the roots and consequently affects the root architecture (Mounier et al., 2014), mainly lateral root growth, which is critical for water and nutrient uptake (Forde, 2014). For instance, *Arabidopsis* with 25.0 μM Cu in the nutrient solution exhibited moderate reduction in the primary root growth and absence of lateral root growth (Lequeux et al., 2010). However, with 50.0 μM Cu in solution, plants exhibited high density of lateral roots, but with drastic reduction in the elongation of both primary and lateral roots (Lequeux et al., 2010). Similarly, *Brassica* also exhibited reduced lateral root growth when exposed to Cu excess in the root medium (Feigl et al., 2013). Moreover, lateral roots are the location of *NRT1.2* (Wang et al., 2012), and a down-regulation of this gene was observed under Cu excess (Fig. 4).

*NRT2.4* is classified as a HAT gene like *NRT2.1* and *NRT2.2*, but no difference in gene expression was observed under the varying Cu levels in this study, with the exception of plants after 72 h with 20.0 μM Cu, where expression was decreased (Fig. 5). *NRT2.4* is expressed mainly in the root epidermis, whereas *NRT2.1* is expressed both in root cortex and epidermis (Kiba et al., 2012). In *Arabidopsis* under nitrate starvation, the expression of *NRT2.4* was observed after 3 days, while the enhancement of *NRT2.1* expression was observed after just a few hours (Wang et al., 2012; Fan et al., 2017). Under Cu excess we are also seeing a different response in these genes with *NRT2.1* and *NRT2.2* showing an up-regulation but *NRT2.4* responding later and showing a down-regulation (Fig. 5).

*NAR2.1* encodes a protein that does not play a role as a transporter of nitrate, but is crucial for the activation of the NRT2 transporters (Orsel et al., 2006; Krapp et al., 2014). Both genes operate interdependently and their expression occurs simultaneously (Okamoto et al., 2006; Fan et al., 2017). Furthermore, with 5.0 and 10.0 μM Cu, *NRT2.1* and *NAR2.1* genes were up-regulated, whereas at the highest concentration of Cu both genes were down-regulated (Fig. 5). Indeed, the lower efficiency of the HATs to promote N uptake recovery by plants under Cu excess, as demonstrated by a lower N concentration in the plant tissue (Fig. 3), could be in part explained by the reduced expression of *NAR2.1*, which did not follow the up-regulation of the *NRT2.1* after 15 days (Fig. 7). The nitrate absorption by HATs is totally dependent on *NAR2.1* expression, which encodes a protein that binds with NRT2 family members, with the exception of *NRT2.7* (Fan et al., 2017). The *Arabidopsis nar2.1* mutant exhibited reduced *NRT2.1* protein amounts in the membrane fraction, as well as lower efficiency of nitrate absorption by the HAT when grown in nutrient solution with low concentrations of nitrate (Orsel et al., 2006).

Nitrate taken up by roots does not only depend on the NRT transporters, but also on H^+^-ATPase activity at the plasma membrane to maintain the equilibrium of protons and anions in the cell and energize the process (Camacho-Cristóbal and González-Fontes, 2007). Here we show that only *AHA2* expression was affected by Cu toxicity (Fig. 6). This effect is unlikely to be due to a general increase in ROS production, since it would affect the transcription of other related H^+^-ATPase genes. Our evidence presented in this study indicates that Cu-excess effects in plants are different when compared to other heavy and non-nutrient metals in plants, such as Cd (Maksymiec and Krupa, 2006; Cuypers et al., 2011; Janicka-Russak et al., 2012). Differences in proton pump activity in membrane vesicles of cucumber (*Cucumis sativus* L.) exposed to Cu and Cd have been reported, with the former causing less damage to plants (Janicka-Russak et al., 2012). This would be possible considering that, even though excess Cu or Cd increases ROS accumulation and cellular damage, up to a certain level, Cu also enhances the activity of antioxidant enzymes, such as superoxide dismutase (Cu/Zn-SOD) and catalase, reducing superoxide radical and hydrogen peroxide in cells (Janicka-Russak et al., 2012; Hippler et al., 2016, Hippler et al., 2018).

Besides nitrate transporters, the expression of the gene encoding nitrate reductase (*NR1*) was very sensitive to Cu excess (Fig. 7). The activity of this enzyme has previously been reported to be affected by Cu toxicity in vines (Llorens et al., 2000) and citrus (Hippler et al., 2016, Hippler et al., 2018). In this study, the reduction of NR1 could be a direct effect of Cu excess by increasing either metal active effects or ROS accumulation in the cell, as well as by a reduction of nitrate uptake resulting after down-regulation of the LATs transporters expression (Fig. 5). Overall, this study demonstrates that under elevated Cu, a sequence of mechanisms operates regulating the uptake and assimilation of N into the roots. Although excess Cu reduced the expression of low-affinity transporters of nitrate (*NRT1.1* and *NRT1.2*; Fig. 4) and *NR1* (Fig. 7), we also observed an increase in the expression of the *NRT2.1* gene up to 72 h (Fig. 5). The increase in the expression of HAT genes followed by the reduction of the *NR1* could in fact result in accumulation of nitrate in the root tissue. However, such accumulation appears not to result in sufficient proportions of N assimilated into proteins (Martins et al., 2014; Tegeder and Masclaux-Daubresse, 2017).

The regulation of *TGA1* and *TGA4* by nitrate has been shown to depend on nitrate transport in plants (Alvarez et al., 2014). Therefore, the observed down-regulation of *TGA1* and *TGA4* (Fig. 7) could be explained by a direct effect of Cu excess or indirectly by Cu-dependent down-regulation of nitrate transporters (Alvarez et al., 2014). Even though the expression of *TGA1* and *TGA4* was reduced by the higher concentrations of Cu in the nutrient solution, it did not limit the up-regulation of *NRT2.1* and *NRT2.2* genes in those plants with 5.0 and 10.0 μM Cu (Fig. 5). Interestingly, *TGA1* and *TGA4* are transcription factors induced by nitrate supply that control the expression of the HATs *NRT2.1* and *NRT2.2*, by direct binding to promoters such as the *SLP9* (Alvarez et al., 2014). In addition to the specific deleterious effects that Cu excess causes on the expression of genes related to uptake, transport and assimilation processes of nitrate in roots, these effects could, in part, result from an increase in ROS production and consequently cellular damage.

With high levels of Cu in plant tissues, membrane lipid peroxidation is likely a response caused by the enzyme activity of lipoxygenase (LOX) (Cuypers et al., 2011), as well as by the hydroxyls radicals (OH^−^) produced by the Fenton reaction under Cu excess (Yruela, 2013). In addition, in *Arabidopsis*, it has been suggested that LOXs can give rise to oxylipins, in particular those belonging to the jasmonate family, which likely act as plant signalling of multiple defence responses under metal stress (Maksymiec and Krupa, 2006; Cuypers et al., 2016). In our study, a marked increase in *LOX1* expression in roots was observed with increasing Cu concentrations in the nutrient solution, and levels were highest at 6 and 24 h after plant exposure to the metal, indicating that such oxylipins were in fact associated with Cu-induced responses observed in our study (Fig. 7).

The increase in membrane lipid peroxidation would lead to a decrease in expression of some genes related to nitrate uptake or proton pump functioning. *Brassica juncea* (L.) exhibited down-regulation of genes related to transport and assimilation of N, both nitrate and ammonium, after abiotic stress conditions including cold, heat, osmotic or salt (Goel and Singh, 2015). Notwithstanding, expression of *BjNRT1.2* was specifically down-regulated by temperature, but not by osmotic or excess salt conditions, our data support that such down-regulation is also likely affected by excess Cu.

Elevated Cu generally reduced accumulation of a number of nutrients in the shoot, as seen for P, Ca, Mn, Zn and B (Table 1). However, Fe was increased in the roots. This has been reported for citrus (Hippler et al., 2016) and *Arabidopsis* (Andrés-Bordería et al., 2017). Therefore, excess Cu in the medium probably reduces the transport of Fe from root to shoot leading to an accumulation in the roots. (Table 1). Fe immobilisation in roots of rice and citrus under high levels of Cu, Zn or Cd in the root medium is likely a response of the plant homeostatic mechanism to reduce metal translocation from roots to shoots, by increasing the amount of organic chelators in the cells, such as histidine, glutathione and nicotianamine (Kendziorek et al., 2014; López-Climent et al., 2014; Gratão et al., 2015; Andrés-Bordería et al., 2017).

Therefore, we demonstrated that high levels of Cu in the root medium decreased the uptake and accumulation of N and other nutrients in plants. This resulted from specific effects on a subset of genes studied: nitrate reductase, the low-affinity transporters of nitrate, members of the proton pump family and bZIP transcription factors that regulate the expression of nitrate transporters. The increased expression of high-affinity transporters of nitrate (NRT2 family) under Cu excess that we observed was insufficient to regain nitrogen absorption by roots. In conclusion, the present study demonstrates the regulation of various N processes under Cu-excess and extends our understanding on the role of genes related to nitrate uptake and assimilation in plants. This will contribute to the selection of genotypes more tolerant to potential excesses of Cu in the environment resulting from intensive use of cupric-based pesticides for healthy management of crops.

## Author contributions

FWRH, DMJ, RMB and LEW designed the study. FWRH performed the experiment. FWRH and LEW analysed the data. FWRH, DMJ and LEW wrote the manuscript. All authors revised the manuscript.

## Disclosures

The authors have no conflict of interest to declare.
