# Supplementary material for: Copper excess reduces nitrate uptake by Arabidopsis roots with specific effects on gene expression
Source: J Plant Physiol. 2018 Sep;228:158–65. doi: 10.1016/j.jplph.2018.06.005 (PMC6090090; doi:10.1016/j.jplph.2018.06.005)
Supplement: Supplementary file 1 [file mmc1.pdf]

Copper excess reduces nitrate uptake by *Arabidopsis* roots with specific effects on gene expression

Franz W.R. Hippler[a,b], Dirceu Mattos-Jr[a], Rodrigo M. Boaretto[a], Lorraine E. Williams[b]

[a]Centro de Citricultura Sylvio Moreira, Instituto Agronômico (IAC), Rod. Anhanguera, km 158, CP 04, CEP 13490-970, Cordeirópolis, SP, Brazil.
[b]University of Southampton, Biological Sciences, Building 85, Highfield, Southampton SO17 1BJ, United Kingdom

SUPPLEMENTARY FILE

Supporting Information Table S1. Genes and primers analysed in roots of *Arabidopsis thaliana* grown under varying copper (Cu) concentrations.

Gene	Locus identification[1]	Forward primer (5' to 3')	Reverse Primer (5' to 3')
**Nitrate transporters**			
NRT1.1 (*NPF6.3* or *CHL1*)	AT1G12110	GGCCGTACTTGTTGCCTTGA	CCCATTGGAATACTCGGCTCA
NRT1.2	AT1G69850	AGCTCAGGGAACACACCTTG	ACGCATTGCCCAAAAGAGGT
NRT1.5	AT1G32450	CGAGCATTGACCTGGTGGTA	CGTTCCTCTTCACTCTCGGT
NRT2.1	AT1G08090	CATCAAGGAAGCCTCCGGTT	TGTTGGGTGTGTTCTCAGGC
NRT2.2	AT1G08100	CGACGCTACGGAGCACTATT	GTTGCGTTCCCTTTGTGGAC
NRT2.4	AT5G60770	CACGGAACAAGGGCTGACA	TCATCTTCCGTGGAAGGCAAA
NAR2.1 (*NRT3.1*)	AT5G50200	CGCCACGCGTCCCTAGATA	CTTGGCCTTCCTCTTCTCATTG
**Plasma membrane H^+-ATPase**			
AHA1	AT2G18960	TAGCTAGGCTTAGGGAGCTTCA	CAGTGTAGTGATGTCCTGCTGT
AHA2	AT4G30190	GAGCTGAGATCGCTAGGCTT	CTACACAGTGTAGTGACTGGGAG
AHA5	AT2G24520	AACCCTCCACGGACTTCAAC	CTCTCTCAGCCTGACAACCTC
**Nitrate reductase**			
NR1 (*NIA1*)	AT1G77760	TGGTACGTCGTTGAAATCGC	TCCCTAAGCACAGCTTCAGTT
**bZIP transcription factors**			
TGA1	AT5G65210	GACAAGCGGCTCGAGGATTA	GTTCACGATGTCGAGTTGCC
TGA4	AT5G10030	GGAGTTTGCTGGTGAGTGGA	TCCTTTAGCCGCAAGACAAGA
**Lipoxygenase**			
LOX1	AT1G55020	GATGAGAGGAACGACGACGAG	CGCCTTCACTGCTCGGAAAC
**Housekeeping genes**			
SAND	AT2G28390	GGTGGATGTTGGAGAGCATT	CCAAACAAGAAGTGGATCCC
YLS8	AT5G08290	CTTCTTCCACATCTGCACTCC	CAGTTCACGGGATATTTCAGC

[1] NCBI data base - (http://www.ncbi.nlm.nih.gov)

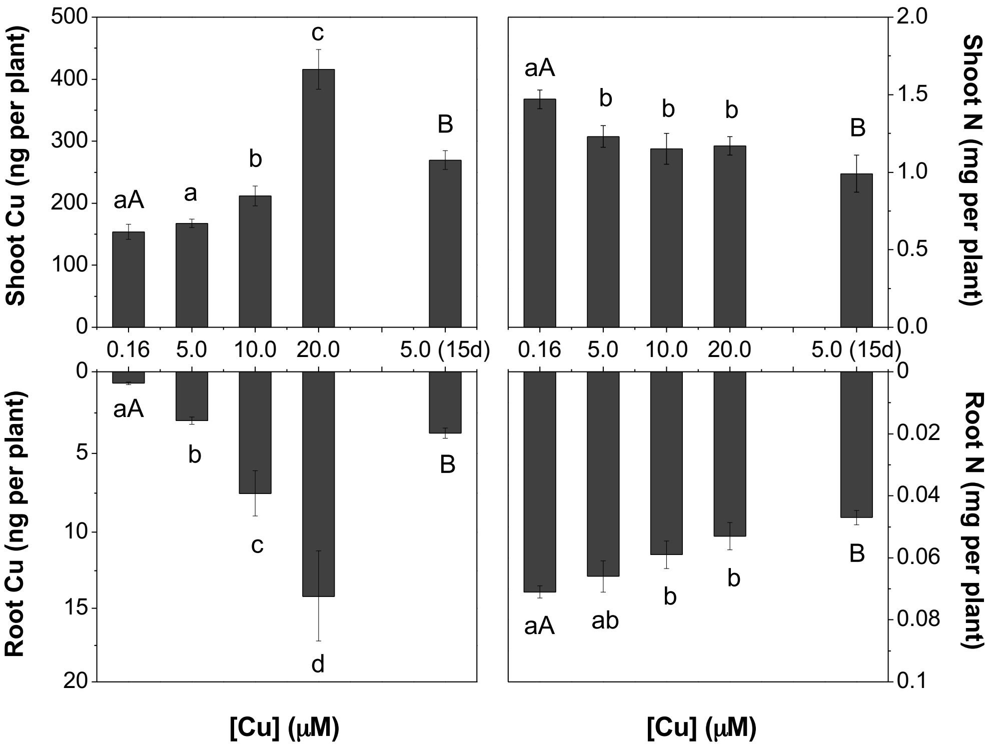

Fig. S1. Elevated copper in the media (Cu) enhanced Cu accumulation but reduced the accumulation of nitrogen (N) in shoot and root of *Arabidopsis thaliana*. Plants were supplied with different Cu levels in the nutrient solution for 72 h or 15 days (15d). Mean (+/- SEM) are shown (n = 3). For the Cu treatments for 72 h different lowercase letters indicate mean values are significantly different among the [Cu] (0.16, 5.0, 10.0 and 20.0 μM) by Tukey's test ($p < 0.05$). For the Cu treatments for 15 days different uppercase letters indicate mean values are significantly different between the [Cu] (0.16 and 5.0 μM) by Tukey's test ($p < 0.05$).

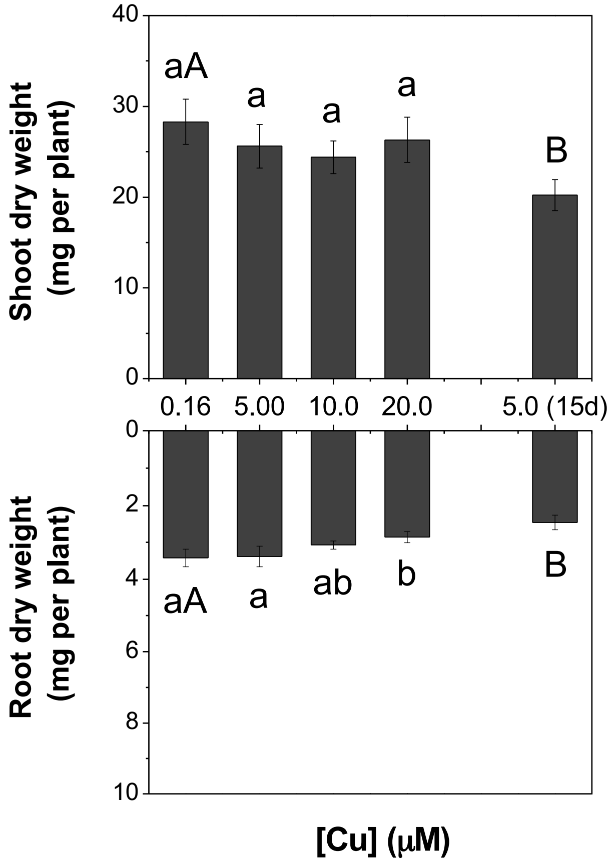

Fig. S2. Elevated copper (Cu) negatively impacts dry weight of shoots and roots of *Arabidopsis thaliana*. Plants were grown under hydroponic conditions for up to 72 h or 15 days (15d). Mean (+/- SEM) are shown (n = 3). For the Cu treatments for 72 h different lowercase letters indicate mean values are significantly different among the [Cu] (0.16, 5.0, 10.0 and 20.0 µM) by Tukey's test ($p < 0.05$). For the Cu treatments for 15 days different uppercase letters indicate mean values are significantly different between the [Cu] (0.16 and 5.0 µM) by Tukey's test ($p < 0.05$). White line in the image represent 2 cm.

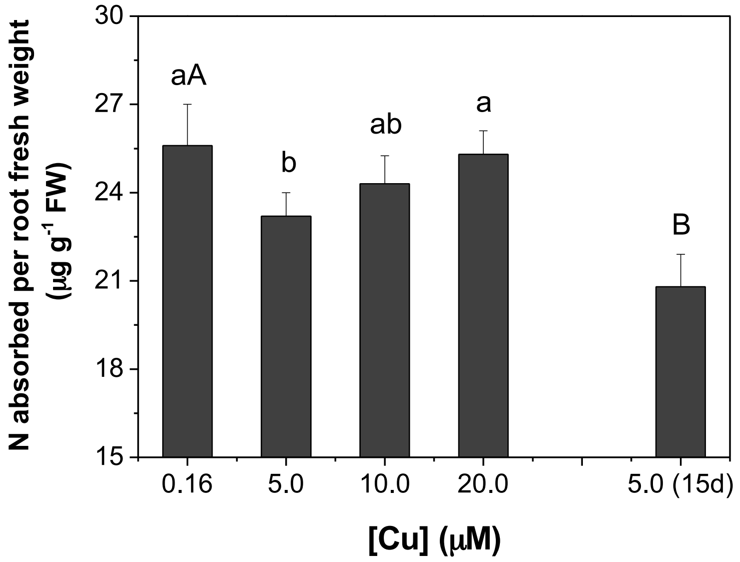

Fig. S3. Elevated copper in the media (Cu) disturbed N absorption per root fresh weight of *Arabidopsis thaliana*. Plants were supplied with different Cu levels in the nutrient solution for 72 h or 15 days (15d). Mean (+/- SEM) are shown (n = 3). For the Cu treatments for 72 h different lowercase letters indicate mean values are significantly different among the [Cu] (0.16, 5.0, 10.0 and 20.0 μM) by Tukey's test ($p < 0.05$). For the Cu treatments for 15 days different uppercase letters indicate mean values are significantly different between the [Cu] (0.16 and 5.0 μM) by Tukey's test ($p < 0.05$).
